# Biological Clocks in the Duodenum and the Diurnal Regulation of Duodenal and Plasma Serotonin

**DOI:** 10.1371/journal.pone.0058477

**Published:** 2013-05-30

**Authors:** Elizabeth Ebert-Zavos, Maria Horvat-Gordon, Alexander Taylor, Paul A. Bartell

**Affiliations:** 1 Department of Animal Science, The Pennsylvania State University, University Park, Pennsylvania, United States of America; 2 Intercollegiate Graduate Program in Physiology, The Pennsylvania State University, University Park, Pennsylvania, United States of America; Florida State University, United States of America

## Abstract

Serotonin in blood plasma is primarily synthesized in the duodenum, as brain derived serotonin does not cross the blood-brain barrier. Because serotonin in the brain and retina is synthesized under the control of a circadian clock, we sought to determine if a circadian clock in the duodenum regulates serotonin synthesis and release in blood. We examined gene expression in the duodenum of chickens at different times of the day and found that the duodenum rhythmically expresses molecular circadian clock genes and genes controlling serotonin biosynthesis, specifically tryptophan hydroxylase, in a light dark cycle (LD). Analysis of the duodenum and blood plasma showed that the amount of serotonin in the duodenum varies across the day and that serotonin profiles in blood plasma are also rhythmic in LD, but were not rhythmic in constant darkness. Because serotonin in the gut affects duodenal nutrient absorption and gut motility, the control of serotonin production in the duodenum by LD cycles could provide an additional mechanism by which the external environment controls nutrient uptake and digestive function. The diurnal regulation of plasma serotonin may also serve as an additional biochemical signal in the blood encoding time and could be used by target tissues to indicate the status of nutrient absorption.

## Introduction

All organisms synchronize their physiology and behavior to the abiotic cycles of their environment. The temporal control of these processes is regulated internally by biological clocks. In birds, circadian oscillators are found within the avian homolog of the suprachiasmatic nucleus, pineal gland, and retina. These clocks function together through a set of inhibitory interactions to form the avian circadian clock system, often described as a “Neuroendocrine Loop” [1]. Within this system, multiple sets of photoreceptors exist to enable entrainment of the system as a whole [1].

At the cellular level, circadian clocks function through the auto­regulatory actions of interlocking positive and negative feedback loops of clock genes. The basic helix­loop­helix­PAS transcription factors circadian locomotor output cycles kaput (clock) and brain and muscle ARNT­like protein (BMAL) heterodimerize and activate E­box (5′­CACGTG­3′) mediated transcription of the negative elements of the loop, in particular Period (*Per*) and Cryptochrome (*Cry)*. The protein products of per and cry translocate into the nucleus and repress BMAL and CLOCK­mediated E­box activation, thereby completing the cycle [for Review see 2, 3]. Genes that are controlled by molecular circadian clocks frequently have E­boxes in their promoter regions and are regulated at the level of transcription by CLOCK and BMAL activation in a rhythmic fashion [4].

In birds, several homologs of clock genes have been found in the retina, pineal, and SCN, including P*er* (*Per2* and *Per3*), Cry (*Cry1* and *Cry2*), *Bmal* (*Bmal1* and *Bmal2*), and *Clock* [5], [6], [7], [8], [9], [10], [11], [12], [13], [14]. Avian homologs of *Bmal1*, *Bmal2*, and *Clock* are rhythmically expressed in the pineal gland with levels highest later in the day. However, in the retina, only bmal1 expression is rhythmic. Similarly, *Per2*, *Per3*, *Cry1*, and *Cry2* are all rhythmic in the pineal [15] whereas only *Per3* and *Cry1* are rhythmic in the retina [16]. In addition, molecular clocks have been described in heart, liver, ovary, and muscle [8], [17], [18]. The avian clock system therefore differs from that of mammals as indicated by the lack of an avian *Per1* homolog and variations in the phasing of some of the clock gene constituents [7], [9], [19], [20]. In mice, clock genes are expressed in epithelial cells and neurons of the myenteric plexus of the colon and are suggested to play an important role in the circadian control of neurotransmitters associated with digestive function [21]. In essence, the gut possesses a time-keeping system that enables the synchronization of gut transit and nutrient absorption to the rhythmic environment and to help coordinate physiological events within the body [22].

### Serotonin

Serotonin is a monoaminergic neurotransmitter derived from tryptophan that is well studied because of its association with mental disorders, most notably depression. Less well studied, however, is the peripheral regulation of serotonin. Most blood borne serotonin is synthesized in the duodenum and has widespread physiological functions beyond those typically associated with mood disorders [23]. Serotonin is unable to cross the blood­brain barrier and, as a result, has varying functions throughout the body, depending on the location of its production and release.

Tryptophan hydroxylase 1 (*Tph1*) is the rate limiting enzyme involved in the synthesis of serotonin in the gut whereas tryptophan hydroxylase 2 (*Tph2*) is the predominant enzyme for serotonin synthesis in the brain and neural tissues [24]. *Tph1* is activated by mucosal stimulation of the gut after meals [25] and is also regulated by low­density lipoprotein receptor­related protein 5 (LRP5) [26]. Serotonin can then either diffuse into enteric nerve endings to promote the digestion and movement of food through the alimentary canal or it can enter circulation [23]. Serotonin is also a direct precursor to endogenous melatonin production and it has been suggested that the gut itself produces melatonin [27], [28]. Interestingly, serotonin and melatonin have opposing effects on gut physiology [29], [30]. Serotonin released into the blood may act as a hormone which, after binding to receptors found on target cells, could potentially provide timing cues to target tissues involving the digestive system and nutritional status. In some tissues, such as the eye and pineal, serotonin is produced under the control of the molecular circadian clock by activating transcription of Tryptophan Hydroxylase [31]. Because of these findings we hypothesized that a circadian clock could control serotonin production in the duodenum and thereby regulate both duodenal and plasma serotonin levels.

## Materials and Methods

### Duodenum collection

White leghorn laying hens (Gallus gallus; approximately 8 months old) were placed in a 16:8 photoperiod, a typical light-dark cycle for raising laying hens, at approximately 20°C, and fed *ad libitum*. Duodenum was harvested from hens that were cervically dislocated 3, 9, 15, and 21 hours after the lights came on, designated as Zeitgeber Time (n = 6 per timepoint). Duodenum was also harvested from birds that were placed in constant darkness for three days at 3, 9, 15, and 21 hours after the lights would normally have come on under their prior photoperiod and is designated as Circadian Time (n = 4 per timepoint). Tissues were frozen on dry ice and stored at −80°C. All animals were utilized in accordance with regulations from Pennsylvania State University's Animal Care and Use Committee (IACUC Protocol #29982).

### RNA extraction

Messenger RNA (mRNA) was extracted from 0.1 g of tissue in 1 mL of Trizol by homogenizing with a roto­stator for approximately 1 minute. Homogenized samples were centrifuged at 12,000×g at 4°C for 5 minutes and the mRNA was removed using an RNeasy kit (Qiagen; Valencia, CA). The supernatant was removed and added to 0.32 mL of chloroform (0.2 mL of chloroform per 1 mL of Trizol). The tubes were then shaken vigorously by hand for 15 seconds, incubated at room temperature for 3 minutes, and centrifuged at 12,000×g for 15 minutes at 4°C. The upper (aqueous) phase was carefully transferred to a fresh 2 mL tube. An equal quantity of 70% ethanol was added to the sample and vortexed. The sample (700 μL) was then transferred to the Qiagen RNeasy column and centrifuged for 30 seconds at 9,000×g. The liquid from the collection tube was discarded and the previous step repeated using the remaining sample. DNaseI solution (10 μL DNaseI and 70 μL RDD buffer) was added to the column and allowed to sit for 15 minutes at room temperature. RWI buffer (700 μL) was then added to the column. The column was centrifuged for 30 seconds at 9,000×g at room temperature, transferred to a new 1.5 mL tube, and centrifuged again at 15,000×g for 1 minute to remove any remaining ethanol. The RNA was eluted into a 1.5 mL collection tube using 35 μL RNase free water by centrifugation for 1 minute at 9,000×g. The sample was checked for concentration and quality of RNA with a spectrophotometer (Nanodrop; Waltham, MA) and then stored at −80°C until needed.

### cDNA synthesis

Complementary DNA (cDNA) was synthesized by combining RNA (1 μg), 1 μL RNase Out (Invitrogen; Carlsbad, CA) and 1 μL random hexamers (New England Biolabs; Ipswich, MA) and heating at 70°C for 10 minutes in a thermocycler (BioRad; Hercules, CA) then immediately cooling on ice. To each tube, 2 μL 10× reaction buffer, 2 mM dNTP mix, and 1 μL M­MLV Reverse Transcriptase (New England Biolabs) were added to make the total reaction volume 20 μL and incubated at 42°C for 60 minutes, followed by heating at 70°C for 15 minutes. After heating, 30 μL of nuclease free water was added to bring the volume to 50 μl and then stored at −80°C until use.

### qRT­PCR

Real time PCR (qRT­PCR) was performed to determine the amount of mRNA of *Per2*, Per3, *Clock*, *Bmal1*, *Bmal2*, *Cry1*, *Vip* and the genes that encode for Tryptophan Hydroxylase, *Tph1* and *Tph2*. A standard reaction volume of 20 μL was comprised of 10 μL SYBR Green Lo Rox Super Mix (Quanta), 6.3 μL water, 0.6 μL of forward primers (Invitrogen; Carlsbad, CA), 0.6 μL reverse primers (Invitrogen), and 2.5 μL of cDNA (50 ng/reaction). Control samples without template and samples without reverse transcriptase were included in each qRT­PCR experiment. Each sample was added to a 96­well plate on ice, covered, and briefly centrifuged at 200 rpm at 4°C. Samples (minus controls) were run in triplicate on the plates using an Opticon II thermocycler (BioRad). Cyclophilin was used as a reference to determine relative gene expression levels, as it is expressed constantly across the day. For each sample, a cycle threshold (Ct) was estimated from the number of cycles required for fluorescence to exceed the background level, and relative gene expression was determined using the formula: ΔΔC(t) =  {C(t)target gene­C(t)Cyclophilin}×time­{C(t)target­ C(t)Cyclophilin [32]. Fold changes were normalized to levels at ZT3, which were arbitrarily set at 1. The following primer sets were utilized in our experiments: *Cyclophilin* (For:GCAAGCAGATCACCATTTCCA; Rev:CGGAATGTCAGGCGTTAAGAC); *Per2* (For:CCCCAGTAGTTGGTGCTCACTT; Rev: GACTGGTGAGCGATACAACACTTT); *Per3*(For:CAGAATGGAAACGATCAGCCTAT; Rev: TCGGGAGAAAACAGGAAGCA); *Bmal1* (For:TTCCCACAGCTTGCAGCTT; Rev: TTTTGGGCCGCCTTCTC); *Bmal2* (For:GAAGCAGAAGTTCTGGAGACTTCAG; Rev: CCACCGAGGAGAGGCTCAT); *Clock* (For:CGTGTGGAGCGGTAATGGT; Rev: GGGCAGCAAAAGTGGGATAA); *Cry1* (For:CCGGGAAACGCCCAAA; Rev: TGCTCTGCCGCTGGACTT); *Vip* (For:CGAAGCCAGGAAGAGTTAAATCCA; Rev: CGTGGCTCAGCAGTTCATCTACA); *Tph1* (For:TGCAAGCAAGAGGGACAGCTTA; Rev: TGCAGGTGACCTTTGGATCA); and *Tph2* (For:ACAGTGAGACCGGTTGCTGGAT; Rev: GATCCGAGCCGTGTCGTACATA).

### HPLC

High­pressure liquid chromatography (HPLC) was used to determine concentrations of serotonin in the duodenum and plasma over the course of the day. Blood samples were collected from the brachial vein of laying hens 3, 9, 15, and 21 hours after lights on (n = 6 per timepoint) using capillary tubes coated with heparin sulfate (125 IU/mL) as an anticoagulant. Another group of birds were placed in constant darkness for three days and blood was collected from these birds 3, 9, 15, and 21 hours after the lights (n = 4 per timepoint) would have come on under their original photoperiod. Blood was placed in heparinized microcentrifuge tubes on ice, centrifuged at 300×g at 4°C for 20 minutes, and the plasma removed. Plasma, (200 μL) and 200 μL of extraction buffer (160 μL of 0.1 M HCLO4, 20 μL of 2.22 mM butylated hydroxytoluene and 20 μL of 0.07 mg/mL methyl­serotonin) were added to a microcentrifuge tube and vortexed. Methyl­serotonin is degraded in a similar manner as serotonin, but has a slightly longer elution time and was used as an internal control. Consequently, the extraction efficiencies and degradation of endogenous serotonin in the samples can be determined by ratios of remaining methyl­serotonin. The solution was then kept on ice for 1 hour to precipitate the protein from the mixture, centrifuged for 12000×g for 20 minutes at 4°C, and the supernatant was removed and filtered through a 0.22 μ Spin­X filter (Corning; Corning, NY) at 2500×g for 5 minutes at 4°C. Duodenal samples (n = 6 per timepoint) were extracted in a similar manner except that 0.1 g of duodenal tissue was sonicated on ice for 1 minute in 1 ml of the extraction buffer.

Extracted samples (10 μl) were separated using a Hypersil Gold a Q column (150×4.6 mm, 5 μm particle size; Thermo Scientific; Waltham, MA) at a flow rate of 0.8 ml/minute. A Hypersil Gold a Q drop­in guard cartridge (10×4.6 mm, 5 μm particle size; Thermo Scientific; Waltham, MA) was used as a pre­filter. The amount of serotonin in the samples was detected using an electrochemical detector (Antec; Frement, CA) with a glass fiber electrode set at +400 mV. The mobile phase consisted of: 97% 20 mM KH_2_PO4 and 20 μM Na_2_EDTA with a pH of 3.0 and 3% MeOH. The column and electrochemical detector were kept at a constant temperature of 30°C. The electrochemical signal from each sample was compared to the signal from a known standard curve to determine the amount of serotonin in each sample. Each sample was run in triplicate.

### Statistical Analysis

Differences among clock gene expression and serotonin content at different time points were evaluated using cosinor analysis to determine rhythmicity and One-way ANOVA with Tukey's post-hoc comparison to determine what time of day production of gene transcripts or serotonin content was different.

## Results

**Figure 1:**
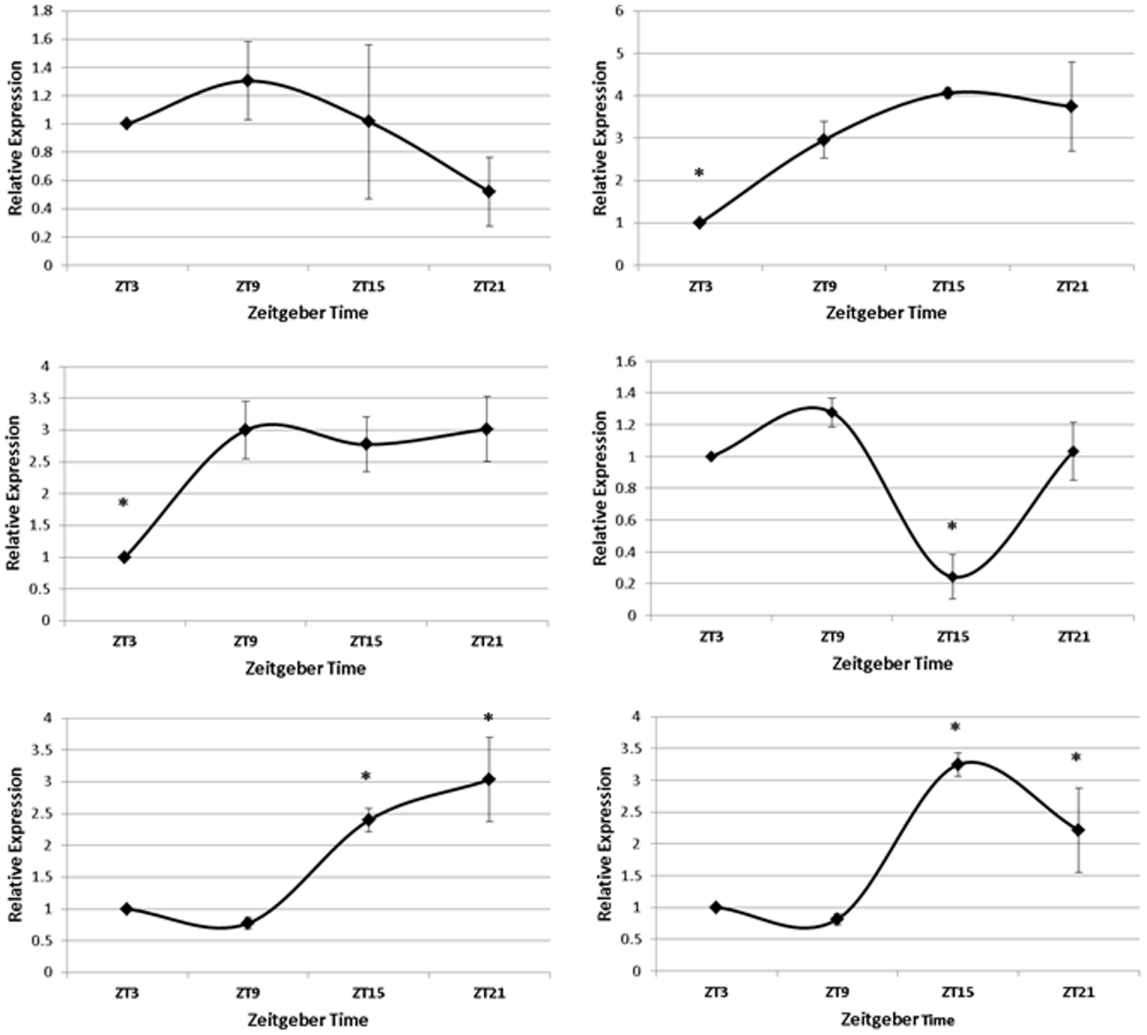
Gene Expression profiles of A) *Cry1*, B) *Bmal1*, C) *Bmal2*, D) *Clock*, E) *Per2*, and F) *Per3* from duodenum of laying hens (n = 6). Asterisks denote time of day when gene expression levels were significantly different from gene expression levels at other times of the day (p<0.05), as determined by One-way ANOVA with Tukey's post-hoc analysis.

The expression profiles for the clock genes *Per2, Per3, Clock, Bmal1,* and *Bmal2* were rhythmic over the course of the day in the duodenum, as determined by COSINOR analysis (Figure 1; p<0.05). The clock gene *Cry1* was found to be not rhythmically expressed in duodenum. *Clock* was most highly expressed just prior to lights-off and levels of *Clock* remained high during lights out. *Bmal1* expression was lowest at the beginning of the day, increased at mid-day and remained high throughout the night. *Bmal2* expression was low during the day and increased right before lights off and stayed high during the night.

**Figure 2:**
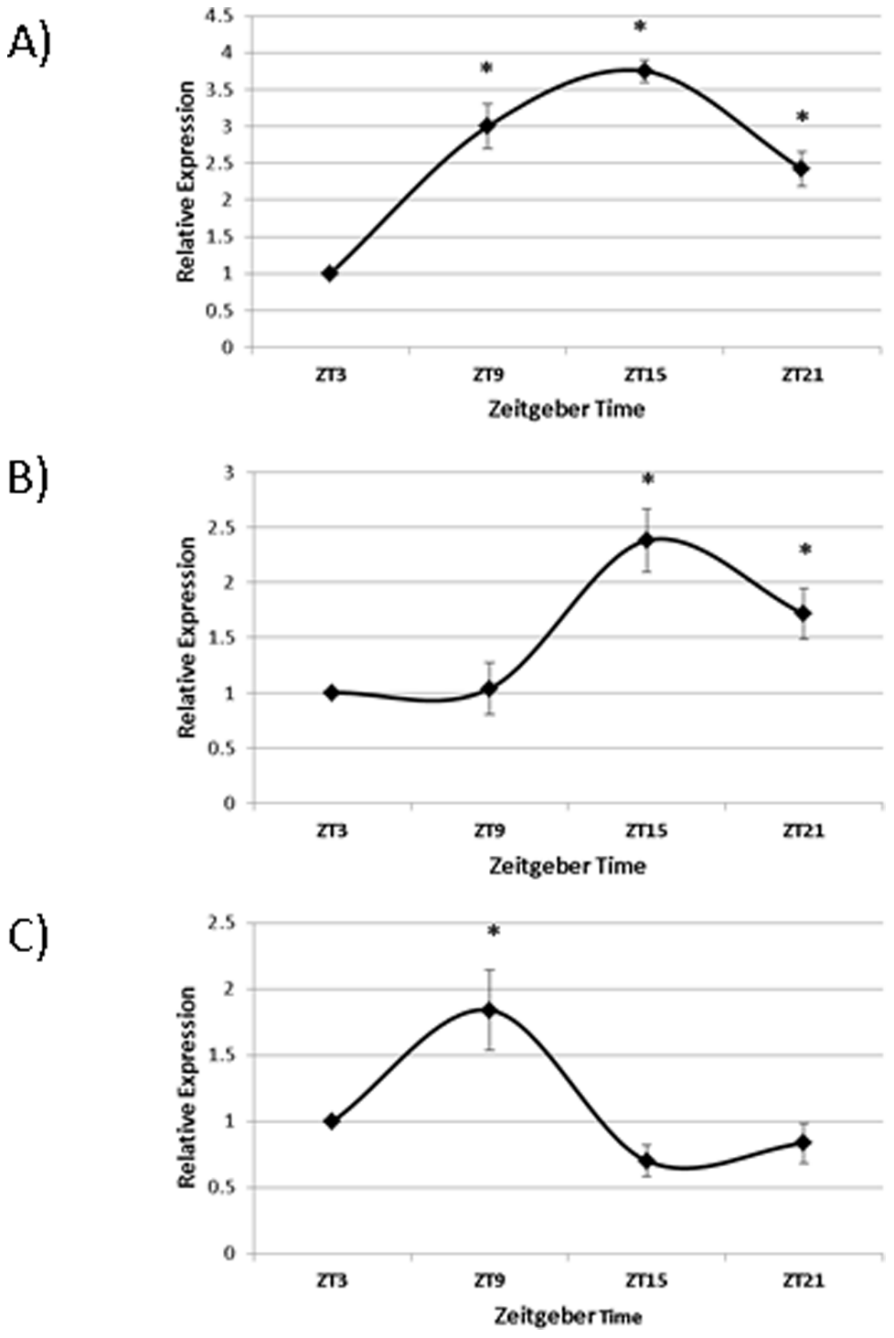
Gene Expression profiles of A) Tryptophan Hydroxylase 1 (*Tph1*) B) Tryptophan Hydroxylase 2 (*Tph2*) and C) Vasoactive Intestinal Polypeptide (*Vip*) from laying hen duodenum (n = 6). Asterisks denote time of day when gene expression levels were significantly different from gene expression levels at other times of the day (p<0.05), as determined by One-way ANOVA with Tukey's post-hoc analysis.

*Per2* levels greatly decreased in expression late during the day before lights out while remaining relatively constant throughout the rest of the 24-hour period. *Per3*expression fluctuated over the course of the day with the highest expression before the onset of darkness. *Cry1*expression did not exhibit much variation through the 24-hour period. Both genes encoding for Tryptophan Hydroxylase, *Tph1* and *Tph2,* and *Vip* expression were rhythmic over the course of the day in the duodenum, as determined by COSINOR analysis (Figure 2; p<0.05). *Vip* expression was highest during the middle of the day and was reduced in expression immediately before lights off and remained low during the night. Levels of Tryptophan Hydroxylase 1 and 2 (*Tph1* and *Tph2*) were highest right before darkness and lowest three hours after lights on.

**Figure 3:**
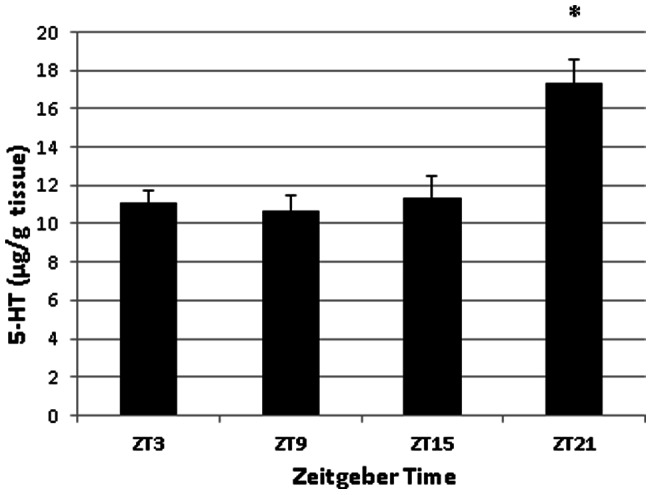
Serotonin content of duodenum from laying hens (n = 6) peaks late in the day at ZT 21 in a 16:8 LD cycle. Asterisk denotes time of day when serotonin levels were significantly different from serotonin content at other times of the day (F(3,28)  = 10.75, p<0.001), as determined by One-way ANOVA with Tukey's post-hoc analysis.

**Figure 4:**
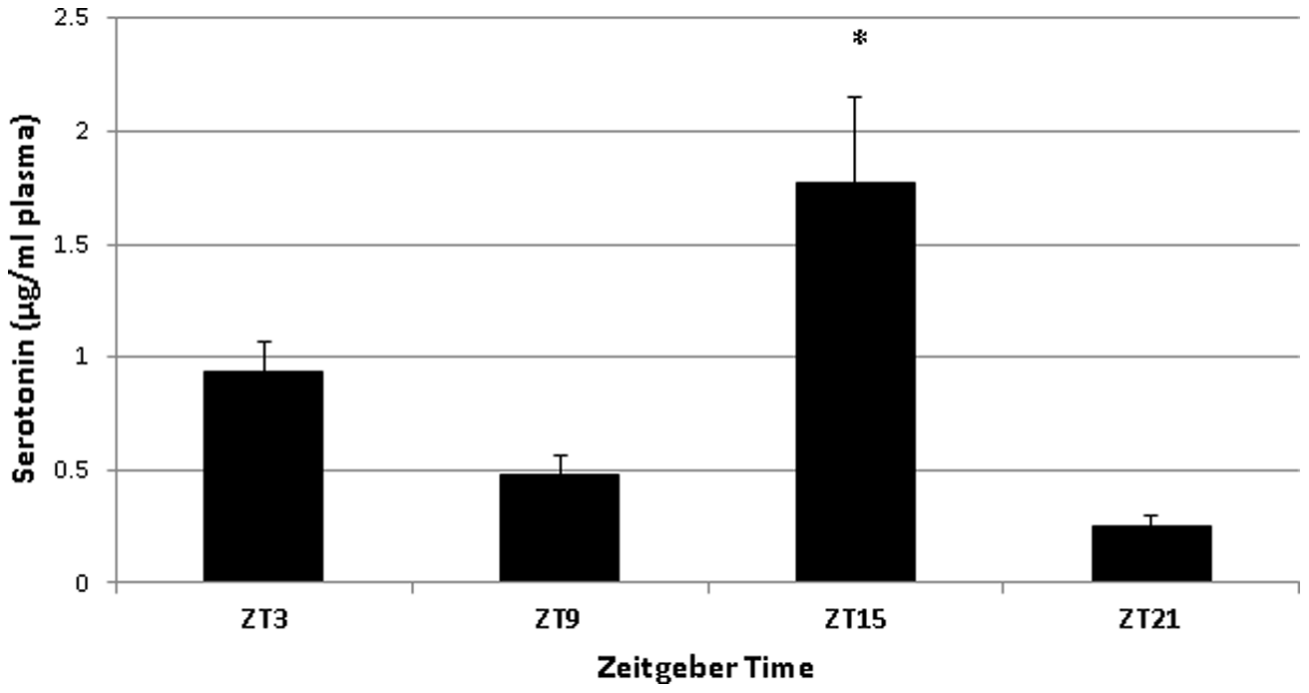
Serotonin content of blood plasma from laying hens (n = 6) in a 16:8 LD cycle. Asterisk denotes time of day when serotonin levels were significantly different from serotonin content at other times of the day (F(3, 18)  = 8.38, P<0.005), as determined by One-way ANOVA with Tukey's post-hoc analysis.

**Figure 5:**
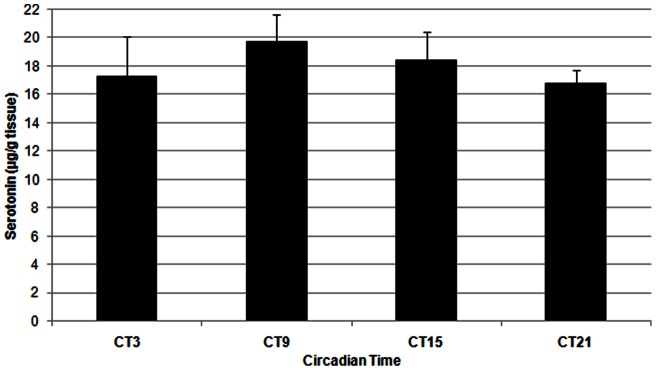
Serotonin content of duodenum from laying hens (n = 4) in constant darkness. Serotonin levels were not significantly different across the day (F(3, 12)  = 0.43, p = 0.739), as determined by One-way ANOVA.

**Figure 6:**
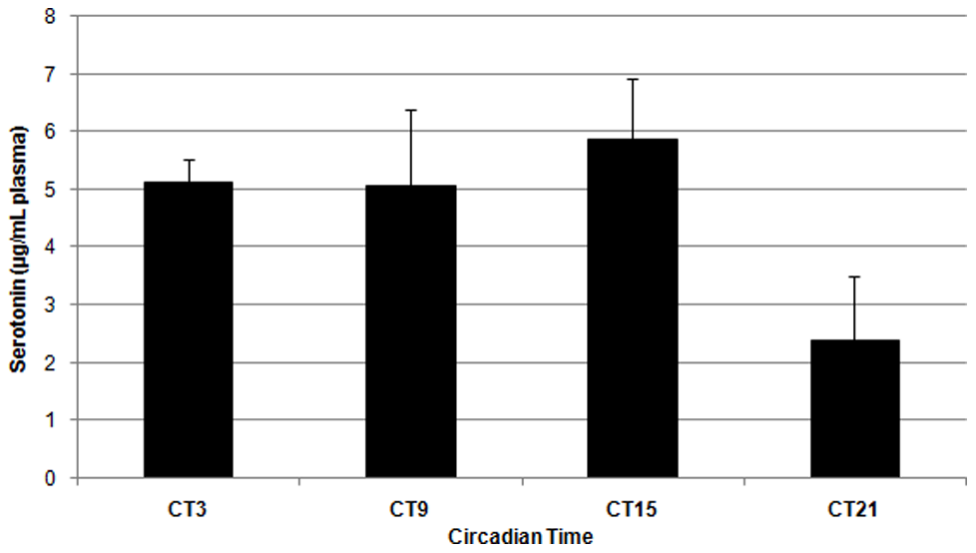
Serotonin content of blood plasma from laying hens (n = 4) in constant darkness. One sample collected at ZT 21 was lost during processing. Serotonin levels were not significantly different across the day (F(3, 11)  = 1.83, p = 0.200), as determined by One-way ANOVA.

Serotonin concentrations in the duodenum and in plasma, as determined with HPLC, were similarly found to fluctuate over the course of the day under a 16:8 LD cycle (Duodenum: Figure 3; F(3,28)  = 10.75, p<0.001); Plasma: Figure 4; F(3, 18)  = 8.38, P<0.005). In the duodenum, levels of serotonin remained low during the day and increased during the night. In plasma, serotonin concentrations were similar in profile to duodenal serotonin content, being relatively constant throughout the day and peaking around the time of lights off. Under constant darkness, however, serotonin was not statistically rhythmic in either duodenum or in blood plasma. (Duodenum: Figure 5; F(3, 12)  = 0.43, p = 0.739; Plasma: Figure 6; F(3, 11)  = 1.83, p = 0.200). In plasma we did see a trend towards rhythmicity, but the data were highly variable, indicating that the rhythm had damped out after several days in constant darkness. The levels of serotonin during the subjective day in the duodenum were significantly higher under constant darkness than duodenal serotonin levels under the 16:8 LD cycle (p<0.05; Student's t-test).

## Discussion

The expression of clock gene products, except for *Cry1*, was rhythmic across the light/dark cycle in the duodenum. These data suggest that a molecular clock exists within the duodenum of chickens which could potentially drive the rhythmic expression of genes associated with feeding and digestion, at least under LD cycles. The ability of the duodenum to maintain time may allow it to anticipate the time of daily food intake, thereby optimizing processes required for efficient uptake of nutrients and the movement of alimentary canal contents in a caudal direction. Consequently, disruption of environmental cues, such as those which occur with phase shifts or changes in feeding schedules, may lead to a disruption of normal rhythmicity in intestinal function and possibly lead to the development of gastrointestinal disorders. Indeed, when birds were placed under constant darkness, the content of serotonin in both blood and duodenum became arrhythmic, suggesting that the duodenal clock is particularly sensitive to environmental input. It is possible that the lack of *Cry1* rhythmicity could make the molecular clock in the duodenum less stable and therefore more dependent upon input from light/dark cycles.

Both Tryptophan Hydroxylase 1and 2 (*Tph1* and *Tph2*) were rhythmically expressed in the duodenum. Because *TPH1* and *TPH2* are the enzymes involved in the rate-limiting step in serotonin biosynthesis, and because *Tph1* and *Tph2* transcription is under diurnal control, our findings suggest that the regulation of transcription by LD cycles could be responsible for the rhythmic expression of serotonin observed in the duodenum under LD. Duodenal serotonin content is comprised of two sources: those in enterochromaffin cells, which are synthesized by TPH1, and those in endocrine cells, which are synthesized by TPH2 [33], [34]. Our measurements of duodenal serotonin reflect the content of both origins and stores, although it has been reported that the amount of serotonin in both duodenum and blood which is derived from TPH1 activity is orders of magnitude greater than serotonin derived from TPH2 activity [35]. Based upon the rhythmic expression patterns of both TPH genes under LD and the lack of serotonin rhythmicity under constant darkness, it appears that serotonin stores from both enterochromaffin and enteric neurons are likely driven by the light/dark cycle. Interestingly, we observed plasma serotonin levels to peak earlier than the peak of serotonin levels in the duodenum under LD. This is most likely due to a reduction in serotonin release from duodenal tissues at night, thereby causing greater serotonin accumulation in the duodenum itself. The finding of higher levels of serotonin during the subjective day when birds are placed in constant darkness gives support to this hypothesis. It has been reported that melatonin, which is high at night in duodenum, can inhibit serotonin release from the gut [36], which could explain why serotonin levels peaked earlier in the day in plasma than serotonin levels in the duodenum. VIP, which we found to be rhythmically expressed in duodenum, can also inhibit the release of serotonin from duodenum [37]. This observation would support our findings too, but caution must be applied as those findings were observed in nocturnal animals and therefore the serotonergic system may be regulated differently than that of diurnal birds. Taken together, however, these works suggest a mechanism by which the high amounts of serotonin in duodenum at night could be regulated. Indeed, our findings of higher serotonin levels in the duodenum of birds during the subjective day than those of birds under LD cycles during the daytime would further suggest that light may promote the release of serotonin from the duodenum.

Our data demonstrate that a molecular clock exists within the duodenum that could regulate the transcriptional machinery required to produce peptides and hormones within the duodenum that could be used to control digestive function under LD cycles. Additionally, our findings suggest that the regulation of plasma serotonin by LD cycles could represent an additional biochemical signal in the blood encoding time and, as a result, serotonin could be used by target tissues throughout the body to integrate timing cues derived from the digestive system.
